# CKAP4 and mutant p53 cooperatively abrogate cell cycle checkpoint to induce genotoxic resistance in ovarian cancer

**DOI:** 10.1002/ctm2.1476

**Published:** 2023-11-20

**Authors:** Canhua Huang, Wei Zhao, Qian Hao, Jianchun Chen, Lisha Wu, Wenqing Yang, Hua Lu, Yu Zhang, Xiang Zhou

**Affiliations:** ^1^ Fudan University Shanghai Cancer Center and Institutes of Biomedical Sciences Fudan University Shanghai China; ^2^ Department of Oncology Shanghai Medical College, Fudan University Shanghai China; ^3^ Department of Gynecology Xiangya Hospital Central South University Changsha China; ^4^ Gynecological Oncology Research and Engineering Center of Hunan Province Changsha China; ^5^ Department of Neurosurgery Changhai Hospital Naval Medical University (Second Military Medical University) Shanghai China; ^6^ Department of Biochemistry and Molecular Biology and Tulane Cancer Center Tulane University School of Medicine New Orleans Louisiana USA; ^7^ Key Laboratory of Breast Cancer in Shanghai Fudan University Shanghai Cancer Center Fudan University Shanghai China; ^8^ Shanghai Key Laboratory of Medical Epigenetics International Co‐Laboratory of Medical Epigenetics and Metabolism (Ministry of Science and Technology), Institutes of Biomedical Sciences, Fudan University Shanghai China

Dear Editor,

Ovarian cancer is the most lethal gynecologic malignancy, primarily characterized by frequent mutations in the *TP53* gene. While platinum‐based chemotherapy has been widely utilized in the treatment of ovarian cancer, patients often experience relapse due to the development of drug resistance. Cytoskeleton‐associated protein 4 (CKAP4) was found to maintain the tubular network along the endoplasmic reticulum‐to‐Golgi pathway and promote cancer progression through various mechanisms.^1^ In this study, we report the novel finding that CKAP4 contributes to mutant p53 (mtp53)‐mediated cancer progression and drug resistance in ovarian cancer.

First, we identified CKAP4 as a possible mtp53‐interacting protein through co‐immunoprecipitation (co‐IP) and mass spectrometric analysis using an ovarian tumour carrying the mtp53‐S241F mutation (Figure S1).^2^, ^3^, ^4^ A set of reciprocal co‐IP assays were conducted to confirm that CKAP4 could bind to various p53 mutants, but not wild‐type p53 (wtp53) (Figure 1A–E). One possible reason for this could be that missense mutations may cause conformational or structural changes to p53, thereby facilitating their interaction. This interaction was further validated by mapping the mtp53‐binding domain on CKAP4 (Figure S2A,B). In addition, our results showed that CKAP4 and mtp53 were present in both the nucleus and cytoplasm (Figure S2C,D). Although CKAP4 did not affect mtp53 expression (Figure S3A,B), we found that ectopic expression of CKAP4 up‐regulated the expression of mtp53 target genes (Figure 1F‐H), while ablation of CKAP4 reduced the expression of these genes (Figure 1I,J). Consistently, our analysis of TCGA database revealed a positive correlation between the levels of CKAP4 expression and the expression of mtp53 target genes in primary ovarian cancer tissues (Figure S4A–I).

**FIGURE 1:**
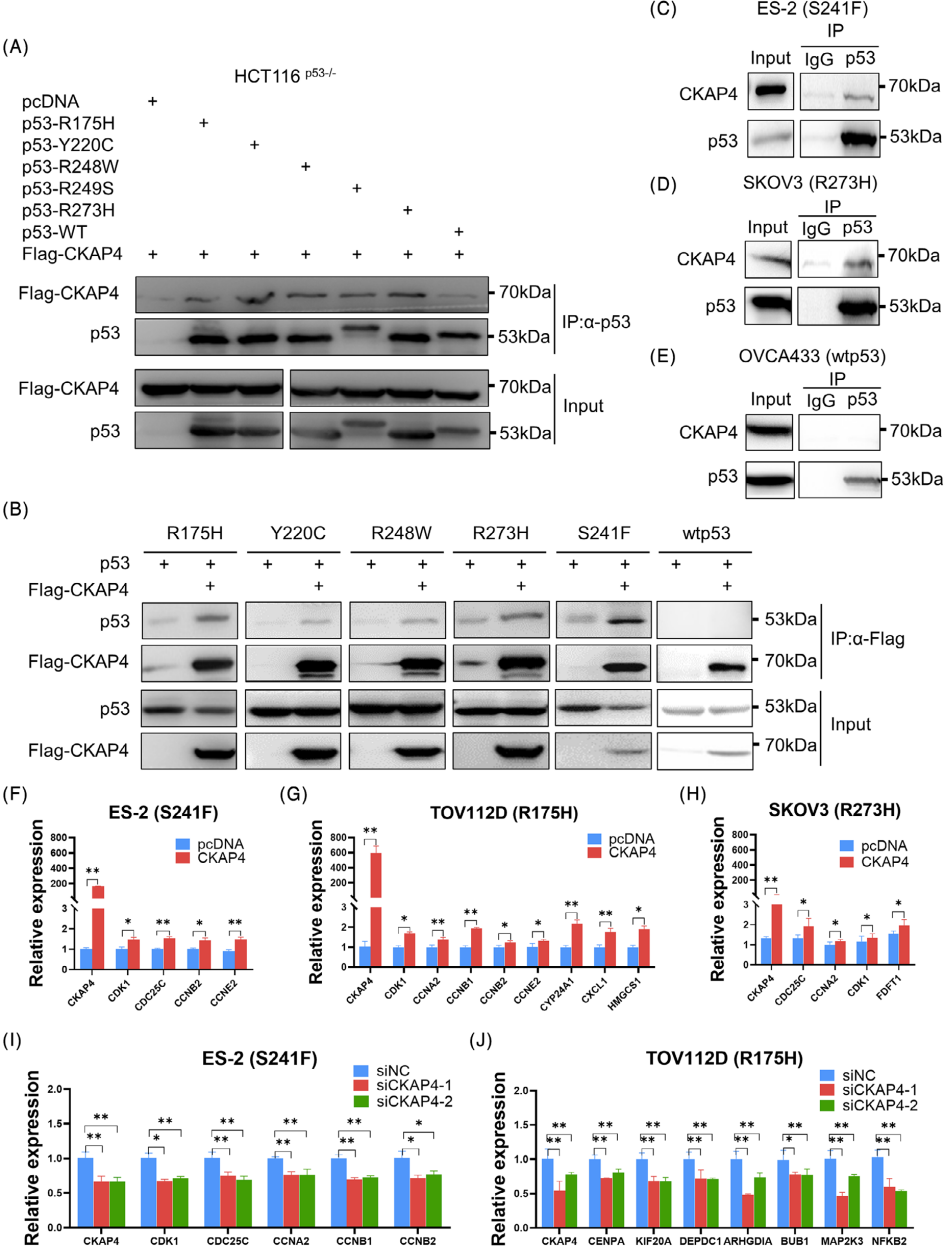
CKAP4 interacts with mutant p53 to increase its target gene expression. (A and B) Flag‐CKAP4 interacts with mtp53s (R175H, Y220C, S241F, R248W and R273H) but marginally binds to wtp53, determined by reciprocal co‐IP assays using the anti‐p53 (A) or anti‐Flag (B) antibody. (C and D) Endogenous interactions between CKAP4 and mtp53s in the ovarian cancer cell lines, ES‐2 (mtp53‐S241F) and SKOV3 (mtp53‐R273H), were detected by co‐IP assays using the anti‐p53 antibody. (E) Endogenous CKAP4 does not bind to endogenous wtp53 in OVCA433 cells. (F–H) Overexpression of CAKP4 upregulates mtp53 target genes in ES‐2 (F), TOV112D (G), and mtp53‐R273H‐stably expressing SKOV3 (H) cells. Cells were transfected with control or CAKP4‐encoding plasmid for 36−72 h, followed by RT‐qPCR analysis. (I and J) Knockdown of CKAP4 represses mtp53 target gene expression in ES‐2 (I), TOV112D (J) cells. Cells were transfected with control or CKAP4 siRNA for 36−72 h, followed by RT‐qPCR analysis. * *p* < 0.05 and ** *p* < 0.01.

To further understand the function of CKAP4 in ovarian cancer, we conducted an analysis using the CPTAC and KM plotter databases. Our analysis showed that both RNA and protein levels of CKAP4 were higher in ovarian cancer tissues than normal tissues (Figure S5A,B). This elevated expression was correlated with poor prognosis of patients with ovarian cancer (Figure S5C–H). Through cell‐based assays, we found that CKAP4 overexpression increased the growth of ovarian cancer cells, while CKAP4 knockdown suppressed their growth (Figure 2A‐F). Consistently, ectopic CKAP4 inhibited ovarian cancer cell apoptosis, whereas CKAP4 depletion promoted apoptosis (Figure 2G‐J). Moreover, the biological function of CKAP4 was investigated in xenograft mouse models. Our results demonstrated that CKAP4 overexpression increased tumour growth rate, weight and size (Figure 3K–M). However, CKAP4 depletion suppressed tumour growth in vivo (Figure 3N–P). Furthermore, CKAP4 overexpression promoted ovarian cancer cell migration (Figure S6A,B), whereas CKAP4 depletion inhibited their migration (Figure S6C–E), probably because CKAP4 regulated metastasis‐associated genes via mtp53 (Figure 1F–J). Lastly, our findings demonstrated that depletion of mtp53 negated the effects of CKAP4 on ovarian cancer cell growth, resistance to apoptosis and migration (Figure S7A–E).

**FIGURE 2:**
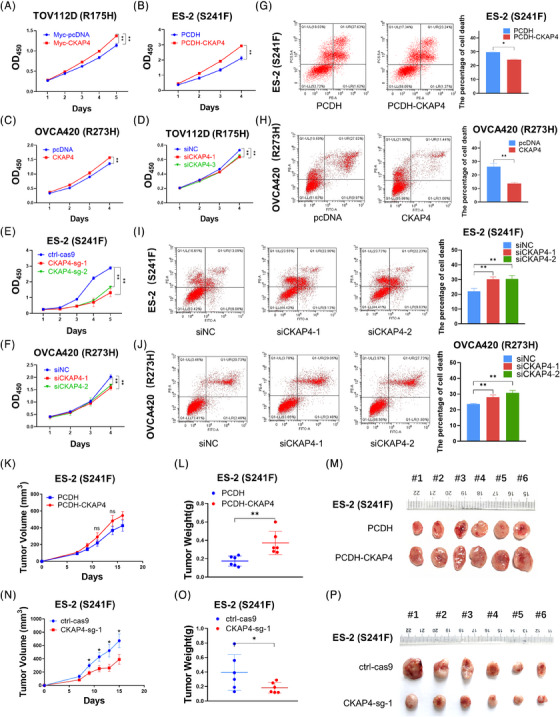
CKAP4 promotes survival and growth of ovarian cancer cells. (A–C) Overexpression of CKAP4 promotes proliferation of TOV112D, ES‐2, and OVCA420 cells. Cells were transfected with control or CAKP4‐encoding plasmid for 12 h, followed by the cell viability assay. (D–F) Knockdown of CKAP4 inhibits proliferation of TOV112D, ES‐2 and OVCA420 cells. Cells were transfected with control or CAKP4 siRNA for 12 h, followed by the cell viability assay. (G and H) Overexpression of CKAP4 inhibits apoptosis of ES‐2 and OVCA420 cells. Cells were transfected with control or CAKP4‐encoding plasmid and treated with cisplatin for 48 h, followed by the flow cytometry analysis. (I and J) Knockdown of CKAP4 promotes apoptosis of ES‐2 and OVCA420 cells. Cells were transfected with control or CAKP4 siRNA and treated with cisplatin for 48 h, followed by the flow cytometry analysis. (K–M) Stable overexpression of CKAP4 suppresses ES‐2‐derived xenograft tumour growth rate (K), weight (L) and size (M). Data are represented as mean  ±  SD, *n*  =  6. (N–P) CRISPR/Cas9‐mediated depletion of CKAP4 promotes ES‐2‐derived xenograft tumour growth rate (N), weight (O), and size (P). Data are represented as mean  ±  SD, *n*  =  6. * *p* < 0.05 and ** *p* < 0.01.

**FIGURE 3:**
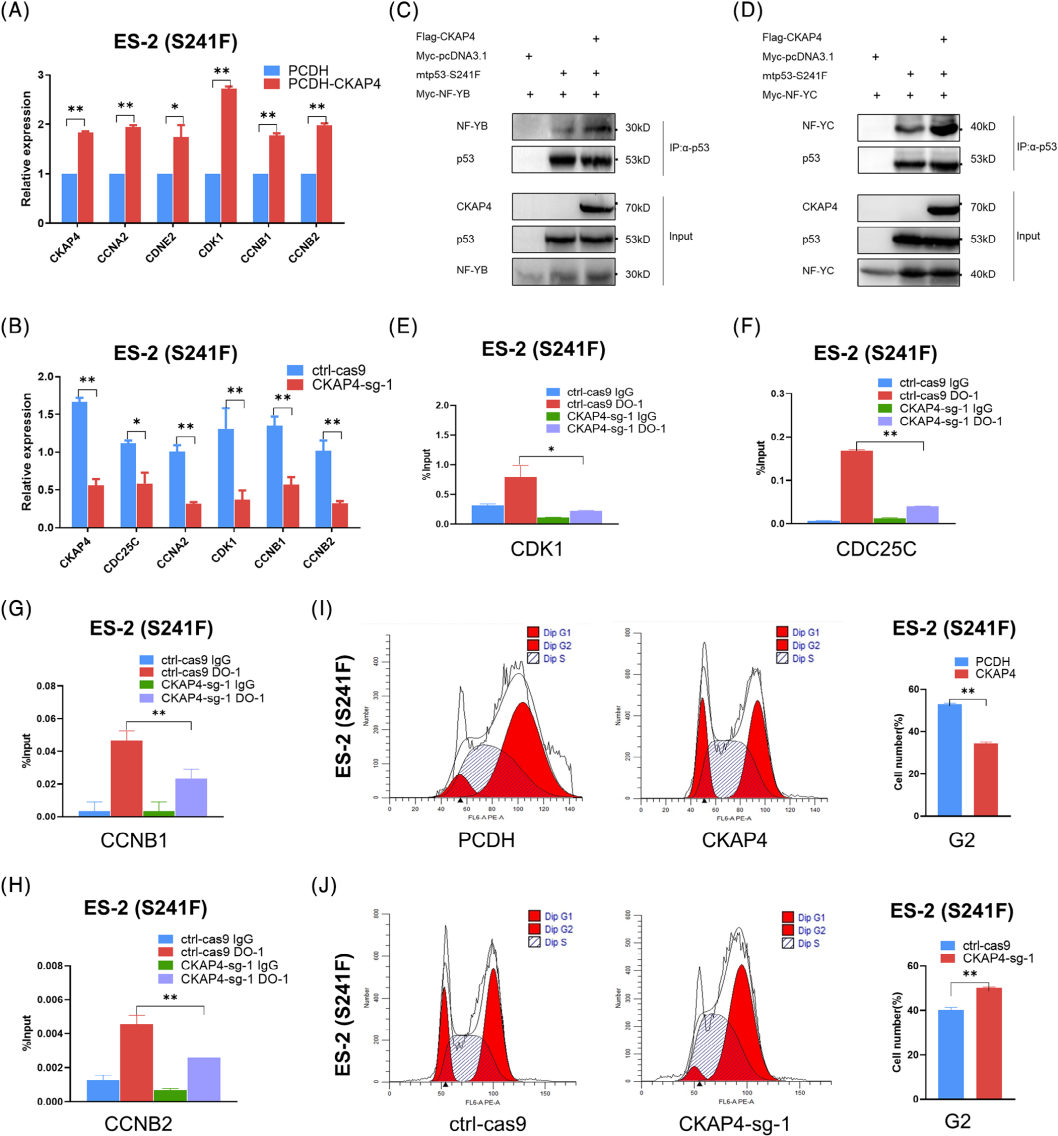
CKAP4 induces the G2/M cell cycle progression via the mutant p53‐NF‐Y axis. (A) Overexpression of CKAP4 increases mRNA levels of cell cycle‐associated genes, such as CDK1, CCNB1, CCNB2 and CCNA2 in xenograft tumours shown in Figure 2. (B) Depletion of CKAP4 reduces mRNA levels of cell cycle‐associated genes in xenograft tumours shown in Figure 2. (C and D) Ectopic CKAP4 enhances mtp53 interaction with NF‐YB (C) and NF‐YC (D). Cells were transfected with the indicated plasmids for 48 h, followed by co‐IP‐IB analysis. (E–H) Depletion of CKAP4 prevents the recruitment of mtp53 on the promoters of CDK1 (E), CDC25C (F), CCNB1 (G) and CCNB2 (H) in response to cisplatin treatment, determined by ChIP analysis. (I) Overexpression of CKAP4 promotes the G2/M cell cycle transition in response to cisplatin treatment. (J) Depletion of CKAP4 induces cell cycle arrest at G2 phase in response to cisplatin treatment. * *p* < 0.05 and ** *p* < 0.01.

We observed that CKAP4 significantly induced the expression of several G2/M checkpoint genes, including CDK1, CDC25C, CCNA2, CCNB1 and CCNB2, which are regulated by the transcription factor nuclear transcription factor (NF)‐Y, as evidenced by the cell‐based results (Figure 1F–J) and in vivo xenograft models (Figure 3A,B). NF‐Y is composed of three subunits, NF‐YA, NF‐YB and NF‐YC, all of which are essential for the binding to the consensus CCAAT motif of eukaryotic promoters.^5^ It was reported that mtp53 gained the oncogenic function by interacting with NF‐Y and boosting its transcriptional activity.^6^ Therefore, we postulated that CKAP4 might regulate cell cycle progression through the mtp53‐NF‐Y pathway. Our findings revealed that CKAP4 overexpression markedly increased mtp53 interactions with NF‐YB and NF‐YC (Figure 3C, D). CKAP4 might serve as a link between mtp53 and NF‐Y by physically interacting with both components. However, further investigation is required to provide more details on the biochemical process. In addition, the chromatin immunoprecipitation (ChIP) assay showed that mtp53 associated with the promoters of various NF‐Y target genes, including CDK1, CDC25C, CCNB1 and CCNB2, in response to DNA damage stress, while CKAP4 depletion hindered these interactions in ovarian cancer cells (Figure 3E–H). Furthermore, we found that CKAP4 overexpression reduced G2 phase accumulation, whereas CKAP4 depletion resulted in cell cycle arrest at G2 phase (Figure 3I,J).

Cisplatin treatment reduces membrane integrity during G2 cell cycle arrest, consequently leading to apoptosis,^7^ and an increase in G2 arrest enhances tumour sensitivity to cisplatin.^8^, ^9^ Thus, we speculated that CKAP4 overexpression could be associated with cisplatin resistance, while targeting CKAP4 might increase ovarian cancer cell sensitivity to cisplatin. Several lines of evidence supported this hypothesis. First, CKAP4 overexpression restored cell growth back to normal levels in the presence of cisplatin (Figure 4A), whereas CKAP4 knockout promoted cisplatin‐induced inhibition of cell growth (Figure 4B). Additionally, CKAP4 overexpression increased the half‐maximal inhibitory concentration (IC50) of cisplatin from 6.503 to 12.15 μM in ES‐2 cells (Figure 4C); conversely, CKAP4 ablation could reduce the IC50 of cisplatin in multiple ovarian cancer cell lines (Figure 4D–F). Furthermore, the colony formation assay again revealed that CKAP4 overexpression induced cisplatin resistance, whereas its depletion increased ovarian cancer cell sensitivity to cisplatin (Figure 4G–I). In agreement with these cell‐based results, our findings demonstrated that CKAP4 depletion augmented the anti‐tumour activity of cisplatin in vivo, as evidenced by the marked reduction of tumour growth rate, weight, and size (Figure 4J–L).

**FIGURE 4:**
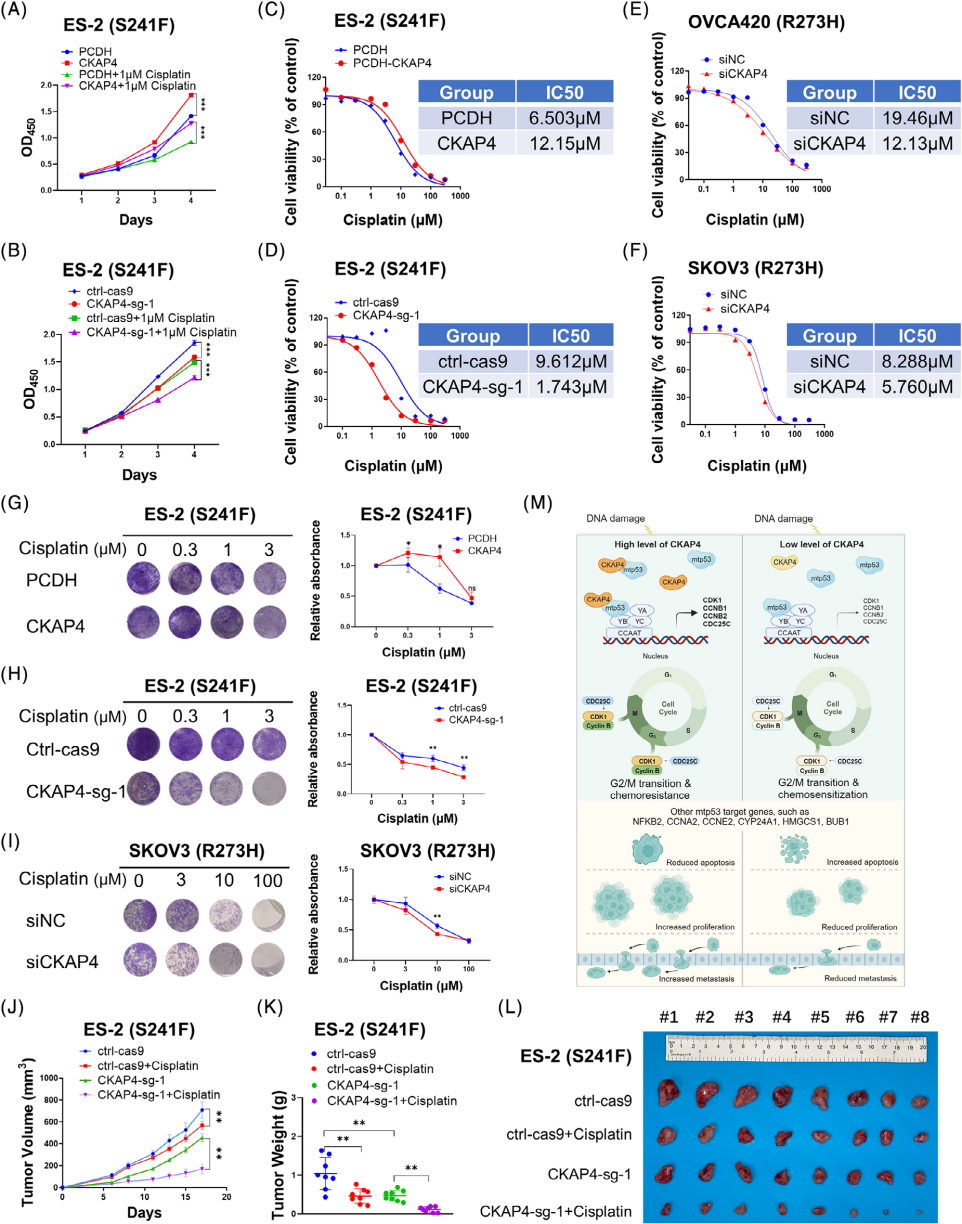
Targeting CKAP4 enhances ovarian cancer sensitivity to cisplatin. (A) Overexpression of CKAP4 restores cell growth following cisplatin treatment. (B) Depletion of CKAP4 enhances cisplatin‐mediated cell growth inhibition. (C) Overexpression of CKAP4 increases ovarian cancer cell resistance to cisplatin, as determined by the IC50 values. (D–F) Depletion of CKAP4 sensitizes ovarian cancer cells to cisplatin, as determined by the IC50 values. (G) Overexpression of CKAP4 increases ovarian cancer cell resistance to cisplatin, as determined by the colony formation assay. (H and I) Depletion of CKAP4 sensitizes ovarian cancer cells to cisplatin, as determined by the colony formation assay. (J–L) Depletion of CKAP4 sensitizes xenograft tumours to cisplatin, as determined by the tumour growth rate (J), weight (K), and size (L). Data are represented as mean  ±  SD, *n*  =  8. (M) Upon DNA damage stress, CKAP4 interacts with mtp53 to enhance NF‐Y‐mediated transcription of cell cycle‐associated genes, leading to accelerated G2/M transition and chemoresistance in ovarian cancer cells (left panel). Depleting CKAP4 impairs the mtp53‐NF‐Y signaling pathway, resulting in cell cycle arrest at G2 phase and increased chemosensitivity in ovarian cancer cells (right panel). * *p* < 0.05, ** *p* < 0.01, and *** *p* < 0.001.

Chemotherapeutic resistance can arise from various mechanisms, one of which is the activation of oncogenic signals.^10^ Mtp53 and NF‐Y signaling pathways were found to coordinately promote chemoresistance in cancer, as mtp53 interacts with the trimeric NF‐Y transcription factor to enhance the expression of multiple genes that regulate cell cycle progression in response to DNA damage stress.^6^ Our study uncovers an oncogenic role for CKAP4 in promoting ovarian cancer progression and chemoresistance. CKAP4 enhances the recruitment of mtp53 to NF‐Y target gene promoters, resulting in the abrogation of the G2/M checkpoint and cisplatin resistance, whereas depleting CKAP4 sensitizes ovarian cancer to cisplatin by triggering cell cycle arrest at G2 phase.

## AUTHOR CONTRIBUTIONS

C.H. and W.Z. conducted most of the experiments and analyzed the data; Q.H. conducted part of experiments; J.C. and H.L. provided important instructions; L.W. and W.Y. analyzed the data; Q.H., Y.Z. and X.Z. conceived, designed and supervised the study and analyzed the data; H.L., Y.Z. and X.Z. wrote and revised the manuscript.

## CONFLICT OF INTEREST STATEMENT

The authors declare no conflict of interest.

## FUNDING INFORMATION

The National Natural Science Foundation of China, Grant Numbers: 82273098, 82072879, 81874053, 82073323 and 82173022; The Natural Science Foundation of Hunan Province, Grant Number: 2021JJ31106; The Reynolds and Ryan Families Chair Fund of Translational Cancer.

## ETHICAL APPROVAL

The present study was approved by the ethics committee of the participating institutions.

## Supporting information

Supporting information

## Data Availability

The data generated or analyzed during this study are included in the article and/or supplementary materials.
