# Supplementary material for: CKAP4 and mutant p53 cooperatively abrogate cell cycle checkpoint to induce genotoxic resistance in ovarian cancer
Source: Clin Transl Med. 2023 Nov 20;13(11):e1476. doi: 10.1002/ctm2.1476 (PMC10659766; doi:10.1002/ctm2.1476)

**CKAP4 and mutant p53 cooperatively abrogate cell cycle checkpoint to induce genotoxic resistance in ovarian cancer**

Canhua Huang^1,2,3,4,#^, Wei Zhao^1,2,3,4,#^, Qian Hao^1,2^, Jianchun Chen^5^, Lisha Wu^3,4^, Wenqing Yang^3,4^, Hua Lu^6^, Yu Zhang^3,4,*^, and Xiang Zhou^1,2,7,8,*^

^1^ Fudan University Shanghai Cancer Center and Institutes of Biomedical Sciences, Fudan University, Shanghai 200032, China

^2^ Department of Oncology, Shanghai Medical College, Fudan University, Shanghai 200032, China

^3^ Department of Gynecology, Xiangya Hospital, Central South University, Changsha 410008, China

^4^ Gynecological Oncology Research and Engineering Center of Hunan Province, Changsha 410008, China

^5^ Department of Neurosurgery, Changhai Hospital, Naval Medical University (Second Military Medical University), Shanghai 200433, China

^6^ Department of Biochemistry & Molecular Biology and Tulane Cancer Center, Tulane University School of Medicine, New Orleans, LA 70112, USA

^7^ Key Laboratory of Breast Cancer in Shanghai, Fudan University Shanghai Cancer Center, Fudan University, Shanghai 200032, China

^8^ Shanghai Key Laboratory of Medical Epigenetics, International Co-laboratory of Medical Epigenetics and Metabolism (Ministry of Science and Technology), Institutes of Biomedical Sciences, Fudan University, Shanghai 200032, China

^#^ Equal contribution

^*^ Correspondence:

Yu Zhang, Email: xyzhangyu@csu.edu.cn

Xiang Zhou, Email: xiangzhou@fudan.edu.cn

**Materials and Methods**

**Plasmids and antibodies**

The Flag-tagged CKAP4-expressing plasmid was purchased from Vigene Biosciences, Inc. The Myc-tagged CKAP4 plasmid was generated by inserting the full-length cDNA amplified by PCR into the pcDNA3.1/Myc-His vector between the XhoI and NheI sites, using the primers in Supplementary Table S1. The plasmids encoding non-tagged mtp53s (R175H, Y220C, S241F, R248W, R249S, and R273H) were previously described.^1^ The anti-Myc (Catalog No. 60003-1, Proteintech), anti-p53 (DO-1, Catalog No. sc-126, Santa Cruz Biotechnology), anti-CKAP4 (Catalog No. ab152154, Abcam), anti-α-Tubulin (Catalog No. 66031-1, Proteintech), anti-Histone-H3 (Catalog No. 17168-1, Proteintech), and anti-GAPDH (Catalog No. 60004-1, Proteintech) antibodies, the secondary antibodies for rabbit (Catalog No. ARG65351, Arigo) and mouse (Catalog No. ARG65350, Arigo), and the light chain-specific secondary mouse antibody (Catalog No. 115-035-174, Jackson) were commercially purchased.

**Cell culture and transient transfection**

Human ovarian cancer cell lines, ES-2, OVCA420, OVCA433, TOV112D, and SKOV3, were cultured in Dulbecco’s modified Eagle’s medium supplemented with 10% fetal bovine serum, 100 U/ml penicillin, and 100 µg/ml streptomycin. SKOV3-R273H stable cell line was described previously.^2^ All cells were cultured at 37°C in an incubator containing 5% CO_2_ and were mycoplasma free and authenticated by the Mycoplasma Detection Kit (Yeasen, Shanghai, China). Cells were seeded in the plates at appropriate density the day before transfection according to the manufacturer’s protocol of the Hieff Trans liposomal transfection reagent (Yeasen). Cells were then collected or split for further experiments 24-36 h post-transfection. Sequences for siRNAs were described in Supplementary Table S2. The proteasome inhibitor MG132 (MedChemExpress, Shanghai, China) was added 4‒6 h before cells were harvested for IP.

**Reverse transcription and quantitative RT-PCR (RT-qPCR) analysis**

Total RNA was isolated from cells using RNAiso Plus (Takara, Japan) following the manufacturer’s protocol. Total RNAs of 0.2–1 μg were used as templates for reverse transcription using PrimeScript RT reagent Kit with gDNA Eraser (Takara, Japan). Quantitative RT-PCR (RT-qPCR) was conducted using TB Green Premix according to the manufacturer’s protocol (Takara, Japan). The primers for CKAP4, p53, GAPDH, and mtp53 target genes, including CDC25C, CDK1, CCNA2, CCNB1, CCNB2,^3^ CCNE2, CENPA, DEPDC1, BUB1,^4^ CYP24A1,^5^ CXCL1,^6^ HMGCS1, FDFT1,^7^ KIF20A, MAP2K3, ARHGDIA,^8^ and NFKB2,^9^ were described in Supplementary Table S3.

**Immunoblotting (IB)**

Cells were harvested and lysed using lysis buffer (50 mM Tris/HCl (pH 7.5), 0.5% Nonidet P-40 (NP-40), 1 mM ethylenediaminetetraacetic acid, 150 mM NaCl, 1 mM dithiothreitol (DTT), 0.2 mM phenylmethylsulfonyl fluoride (PMSF), 10 mM pepstatin A, and 1 mM leupeptin. Equal amounts of clear cell lysate were used for immunoblotting (IB) analysis as described previously.^10^

**Immunoprecipitation (IP)**

Immunoprecipitation (IP) assays were conducted using anti-Flag or anti-p53 antibody as indicated in the figure legends. Briefly, 500–1000 μg of proteins in lysis buffer (the same one as used in IB) were incubated with the indicated antibody at 4 °C for 4–10 h. Protein A or G beads (Santa Cruz Biotechnology) were then added, and the mixture was incubated at 4 °C for additional 1–3 h. Beads were washed 3–6 times with lysis buffer.^11^ Bound proteins and 10% inputs were detected by IB with antibodies as indicated in the figure legends.

**Chromatin immunoprecipitation (ChIP)**

The chromatin immunoprecipitation (ChIP) assay was performed following the manufacturer’s protocol of the Magna ChIP A/G Chromatin IP Kit (Merck) as previously described.^12^ Briefly, cells were crosslinked with 1% formaldehyde and then terminated by adding 10 × glycine. Cells were scraped off the dish and resuspended in ChIP lysis buffer containing the protease inhibitor. The cell pellet was collected and lysed with nuclear lysis buffer. Chromatin was sheared into ∼400-∼900-bp fragments by sonication. The anti-α-p53 antibody or IgG was added to the chromatin mixture rotating at 4 °C overnight. The Protein A/G beads were then added, the mixture was incubated at 4 °C for additional 1–3 h and washed sequentially using low salt wash buffer, high salt wash buffer, LiCl wash buffer, TE buffer and then eluted using the elution buffer. The bound DNA was purified and analyzed by qPCR. The primers used in the ChIP assay were listed in Supplementary Table S4.

**Cell viability assay**

To test the long-term cell survival, the Cell Counting Kit-8 (CCK-8) (Yeasen) was used following the manufacturer's protocol as previously described.^13^ Cell suspensions were seeded at 3000-5000 cells per well in 96-well culture plates at 12 h post-transfection. CCK-8 was added at a final concentration of 10% to the cultures for 2–4 h, and the absorbance of the samples at 450 nm was measured using a Microplate Reader every 24 h for 5 days.

**Flow cytometry analysis**

Cell apoptosis was assessed by the Annexin V-PE/7-AAD Apoptosis Detection Kit (Vazyme, Nanjing, China) following the manufacturer's protocol. After transfection for 48-72 h, cells were harvested, washed with cold PBS, and resuspended in 1 × binding buffer. Next, the cells were stained with Annexin V-PE and 7-AAD and then analyzed by a FC500 MPL flow cytometer (Beckham Coulter, Indianapolis, IN, USA). For cell cycle analysis, cells were harvested and fixed with 70% ethanol overnight. After being washed with cold PBS twice, cells were treated with 250 μl buffer (50 μg/ml RNase A and 0.1% Triton X-100 in PBS) at 37°C for 0.5 h. Then, cells were stained with 250 μl buffer [50 μg/ml Propidium Iodide (PI) (MedChemExpress) and 0.1% Triton X-100 in PBS] for 0.5 h in dark, followed by flow cytometry analysis.

**Cell migration assay**

The cell migration assay was performed as previously described.^1^ In brief, 5-10 × 10^4^ cells suspended in 100 µl of serum-free medium were added to the upper chamber. The lower chambers were filled with the normal culture medium. After culture for 24 h at 37 °C, the cells on the upper surface were scraped and washed away, and the cells on the lower surface were fixed with methanol and stained with 0.1% crystal violet. The number of invaded cells was counted in at least three randomly selected fields under an optical microscope.

**Fractionation of cell components**

Cells were gently scraped in PBS, then resuspended in an appropriate amount of cell lysis buffer A (10 mM HEPES, 10 mM NaCl, 0.1 mM EDTA, 0.1 mM EGTA, 1 mM DTT, 1 mM PMSF, and the protease inhibitor cocktail), followed by vortexing several times and incubation on ice for 20 min. 10% NP-40 was added to the mixture at a final concentration lower or equal to 0.05%, followed by vortexing for 10 seconds at the highest setting. The homogenate was centrifuged at 4° C and the supernatant that contains the cytoplasmic fraction was collected. The pellet was resuspended in 40 μl lysis buffer B (20 mM HEPES, 400 mM NaCl, 1 mM EDTA, 1 mM EGTA, 1 mM DTT, 1 mM PMSF, and the protease inhibitor cocktail) and incubated on ice for 30 min with vortexing every 5 m. The homogenate was centrifuged at 4 °C and the supernatant that contains the nuclear fraction was collected. The cytoplasmic and nuclear fractions were then analyzed by IB.

**Mouse xenograft experiments**

BALB/c nude mice of 4–5 weeks old were purchased from and maintained in the Laboratory Animal Science of Fudan University Shanghai Cancer Center. To evaluate the effect of CKAP4 on tumor growth in vivo, mice were subcutaneously inoculated with 5 × 10^5^ ES-2 cells stably expressing PCDH vector, PCDH-CKAP4, Ctrl-Cas9 or CKAP4-Cas9 per flank. To evaluate cisplatin efficacy, mice of Ctrl-Cas9 or CKAP4-Cas9 group were randomly divided into two groups and administered with PBS or cisplatin (3 mg/kg i.p. every other day) when tumors reached 100 mm^3^. Tumor growth was monitored every other day with electronic digital calipers in two dimensions. Tumor volume was calculated according to the formula: volume = length × width^2^ × 0.5.^14^ Tumors were then harvested, weighed, and subjected to qPCR analysis. The animal protocols were in compliance with ethical guidelines and approved by the Animal Welfare Committee of Fudan University Shanghai Cancer Center.

**Database of cancer patients**

The CKAP4 expression in cancerous and normal samples was compared using the Sangerbox tools (<http://www.sangerbox.com/tool>). The prognosis of cancer patients was analyzed using the Kaplan–Meier survival analysis (kmplot.com).^15^ The correlation of CAKP4 and mtp53s’ target genes was analyzed using GEPIA.^16^ *p* < 0.05 was considered statistically significant, and asterisks represent significance in the following way: * *p* < 0.05, ** *p* < 0.02, and *** *p* < 0.01. Quantitative data are presented as mean ± SD.

**Statistics**

All in vitro experiments were performed in biological triplicate. The Student’s *t*-test or one-way analysis of variance was performed to evaluate the differences between two or more groups. *p* < 0.05 was considered statistically significant, and asterisks represent significance in the following way: * *p* < 0.05, ** *p* < 0.02, and *** *p* < 0.01. Quantitative data are presented as mean ± SD.

**References**

1 Chen Y, Hao Q, Wang J et al. Ubiquitin ligase TRIM71 suppresses ovarian tumorigenesis by degrading mutant p53. Cell death & disease 2019; 10 (10): 737.

2 Huang C, Hao Q, Shi G et al. BCL7C suppresses ovarian cancer growth by inactivating mutant p53. Journal of molecular cell biology 2021; 13 (2): 141-150.

3 Di Agostino S, Strano S, Emiliozzi V et al. Gain of function of mutant p53: the mutant p53/NF-Y protein complex reveals an aberrant transcriptional mechanism of cell cycle regulation. Cancer cell 2006; 10 (3): 191-202.

4 Girardini JE, Napoli M, Piazza S et al. A Pin1/mutant p53 axis promotes aggressiveness in breast cancer. Cancer cell 2011; 20 (1): 79-91.

5 Stambolsky P, Tabach Y, Fontemaggi G et al. Modulation of the vitamin D3 response by cancer-associated mutant p53. Cancer cell 2010; 17 (3): 273-285.

6 Yan W, Chen X. Identification of GRO1 as a critical determinant for mutant p53 gain of function. The Journal of biological chemistry 2009; 284 (18): 12178-12187.

7 Freed-Pastor WA, Mizuno H, Zhao X et al. Mutant p53 disrupts mammary tissue architecture via the mevalonate pathway. Cell 2012; 148 (1-2): 244-258.

8 Bossi G, Marampon F, Maor-Aloni R et al. Conditional RNA interference in vivo to study mutant p53 oncogenic gain of function on tumor malignancy. Cell Cycle 2008; 7 (12): 1870-1879.

9 Scian MJ, Stagliano KE, Anderson MA et al. Tumor-derived p53 mutants induce NF-kappaB2 gene expression. Molecular and cellular biology 2005; 25 (22): 10097-10110.

10 Zhou X, Hao Q, Liao J et al. Ribosomal protein S14 unties the MDM2-p53 loop upon ribosomal stress. Oncogene 2013; 32 (3): 388-396.

11 Zhou X, Hao Q, Zhang Q et al. Ribosomal proteins L11 and L5 activate TAp73 by overcoming MDM2 inhibition. Cell death and differentiation 2015; 22 (5): 755-766.

12 Chen Y, Hao Q, Wang S et al. Inactivation of the tumor suppressor p53 by long noncoding RNA RMRP. Proceedings of the National Academy of Sciences of the United States of America 2021; 118 (29).

13 Wang S, Hao Q, Li J et al. Ubiquitin ligase DTX3 empowers mutant p53 to promote ovarian cancer development. Genes & diseases 2022; 9 (3): 705-716.

14 Zhang Q, Zeng SX, Zhang Y et al. A small molecule Inauhzin inhibits SIRT1 activity and suppresses tumour growth through activation of p53. EMBO molecular medicine 2012; 4 (4): 298-312.

15 Gyorffy B, Lánczky A, Szállási Z. Implementing an online tool for genome-wide validation of survival-associated biomarkers in ovarian-cancer using microarray data from 1287 patients. Endocrine-Related Cancer 2012; 19 (2): 197-208.

16 Tang Z, Li C, Kang B et al. GEPIA: a web server for cancer and normal gene expression profiling and interactive analyses. Nucleic Acids Research 2017; 45 (W1): W98-W102.


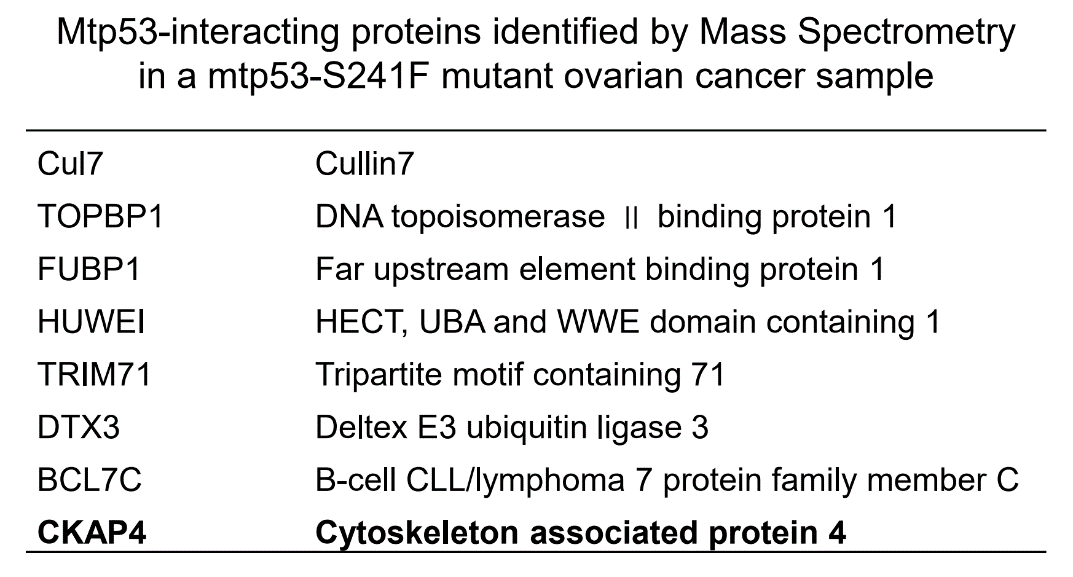


**Supplementary Figure 1.** A list of mtp53-interacting proteins, including CKAP4, which were identified in an ovarian cancer tissue sustaining mtp53-S241F. The other proteins in the list have been previously reported as p53/mtp53-interacting proteins by our group and others.


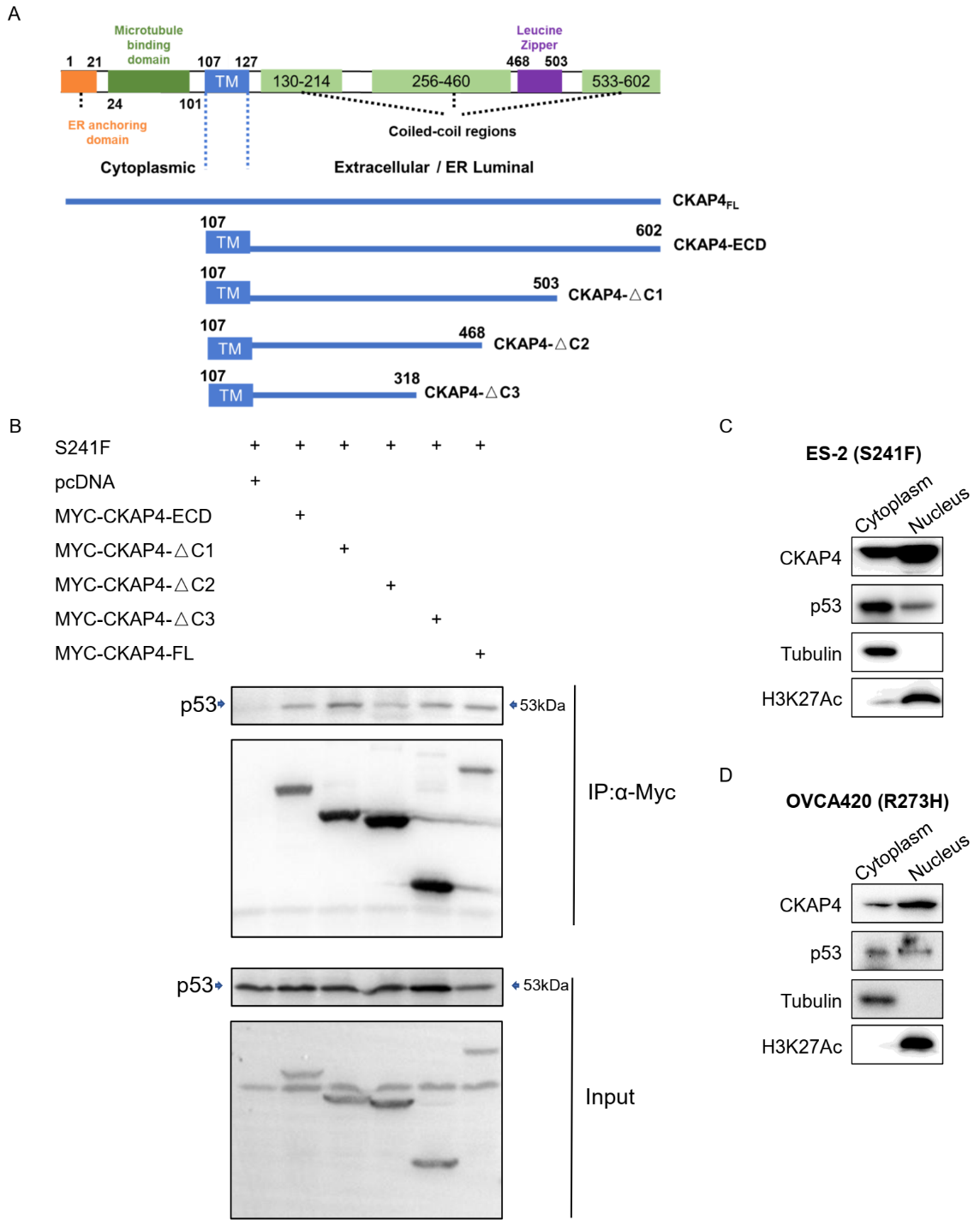


**Supplementary Figure 2. Mapping mutant p53-S241F binding region(s) of CKAP4.** (A) A schematic diagram of CKAP4 fragments. (B) Mtp53-S241F interacts with the amino acids 107-318 of CKAP4. Cells were transfected with plasmids encoding Myc tagged-CKAP4 fragments as indicated, along with the mtp53-S241F-encoding plasmid, followed by co-IP-IB analysis using indicated antibodies. (C, D) The subcellular distribution of CAKP4 and mtp53 proteins in ES-2 and OVCA420 ovarian cancer cells.


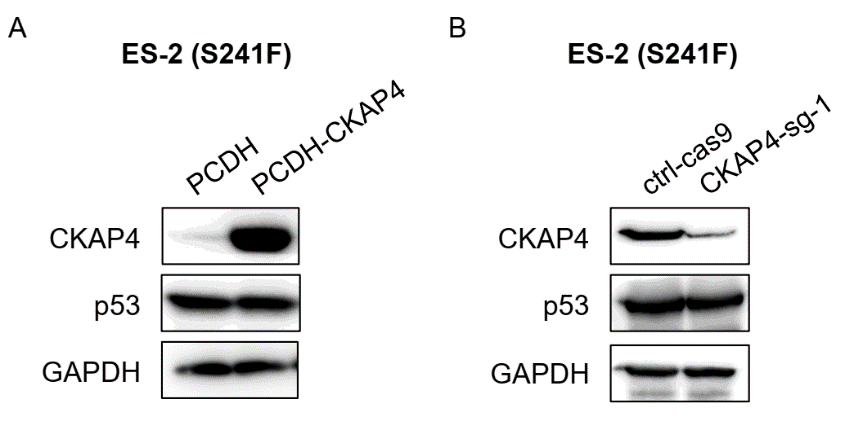


**Supplementary Figure 3. CKAP4 does not affect protein levels of mutant p53.** (A) Overexpression of CKAP4 does not affect mtp53 protein level. (B) Depletion of CKAP4 does not affect mtp53 protein level.


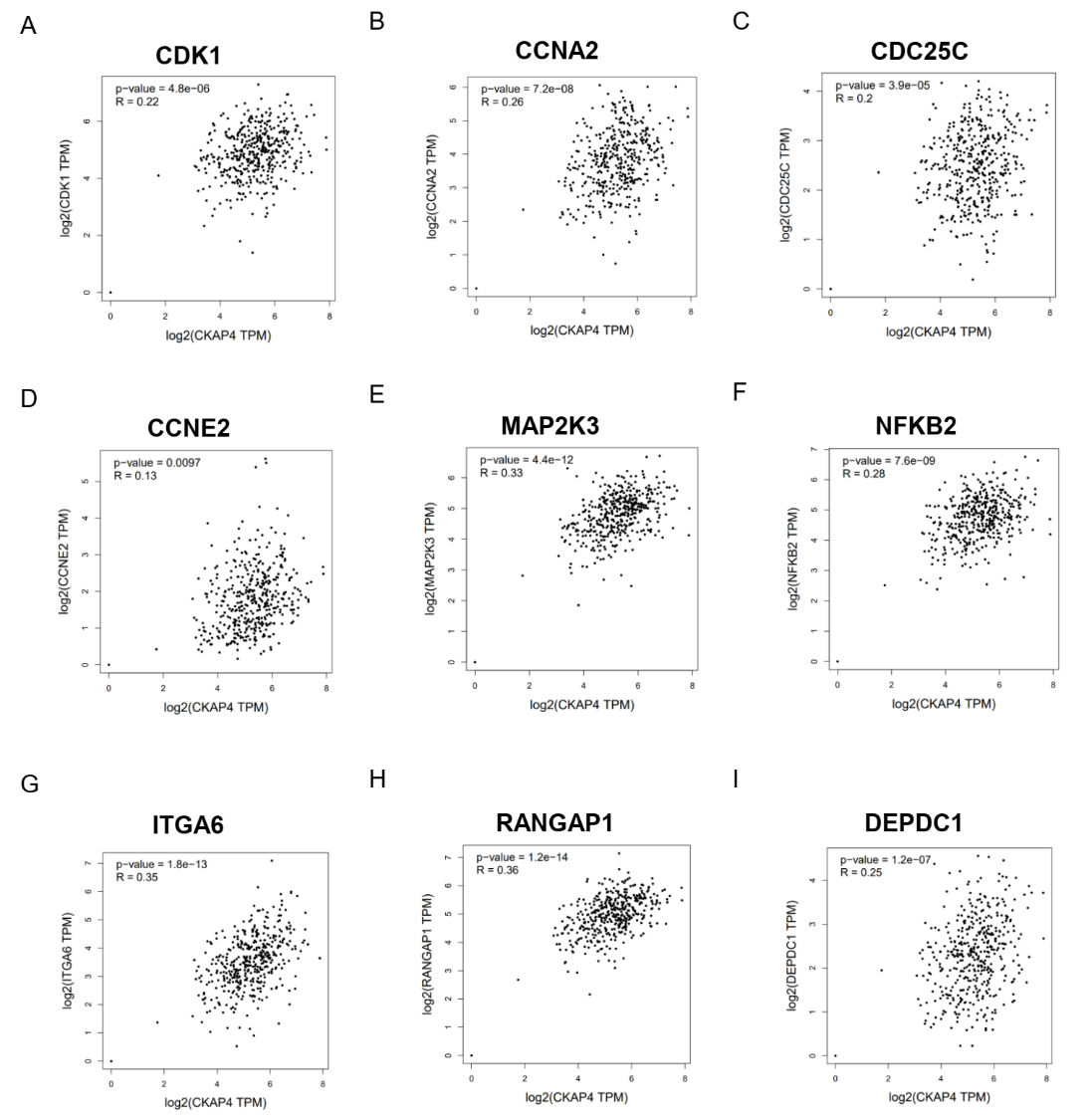


**Supplementary Figure 4. Positive correlation between the expression of CKAP4 and mtp53 target genes.** These target genes are involved in various biological functions, such as proliferation (A-E), survival (F), metastasis (G), and metabolism (H, I), in ovarian cancer.


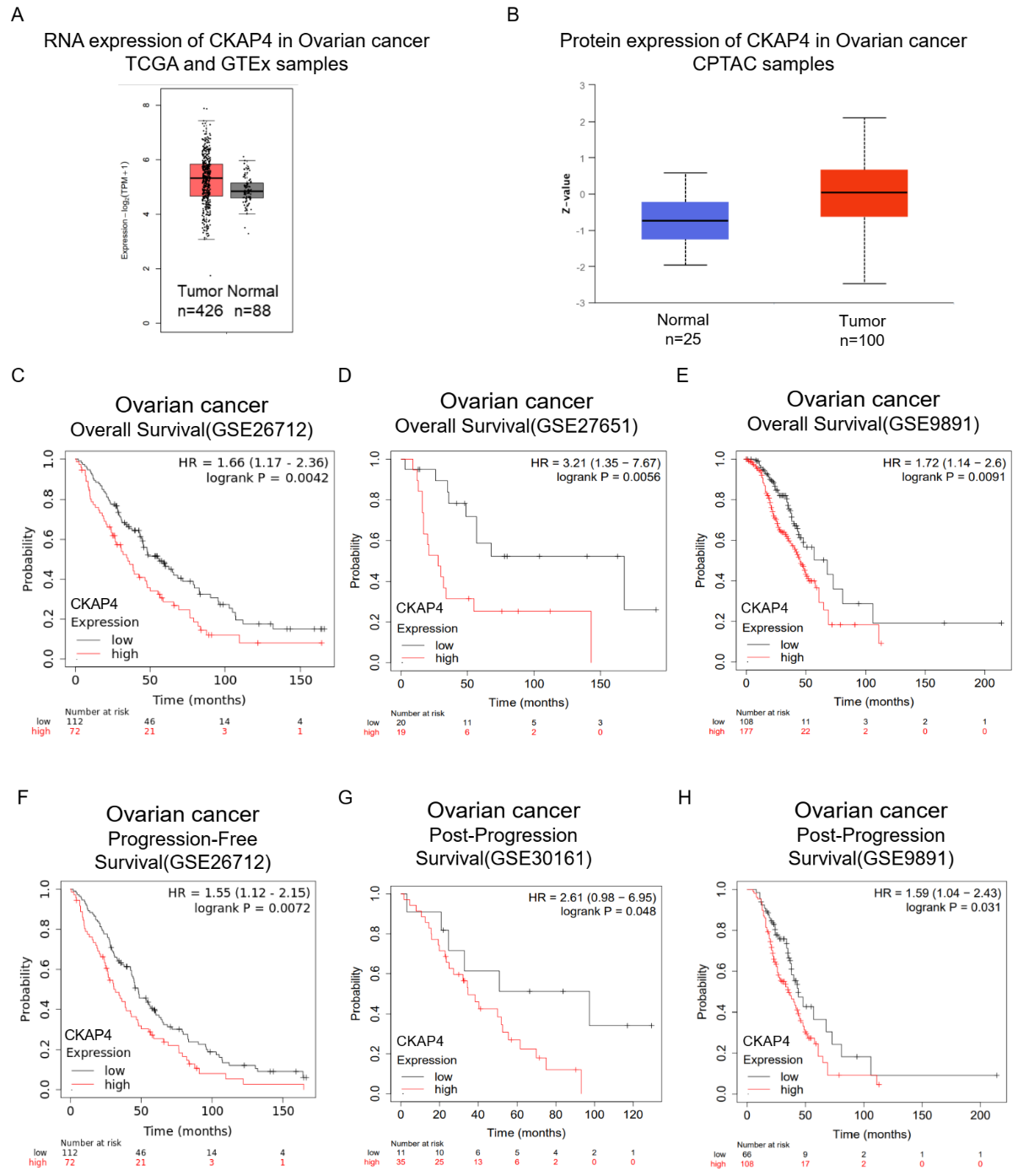


**Supplementary Figure 5. High expression of CKAP4 is associated with unfavorable prognosis in ovarian cancer.** (A, B) The mRNA and protein levels of CKAP4 are upregulated in ovarian cancer compared to normal tissues. (C-H) The Kaplan–Meier analysis reveals that higher CKAP4 expression is correlated with poor overall survival (C, D, E), progression-free survival (F), and post-progression survival in ovarian cancer (G, H).


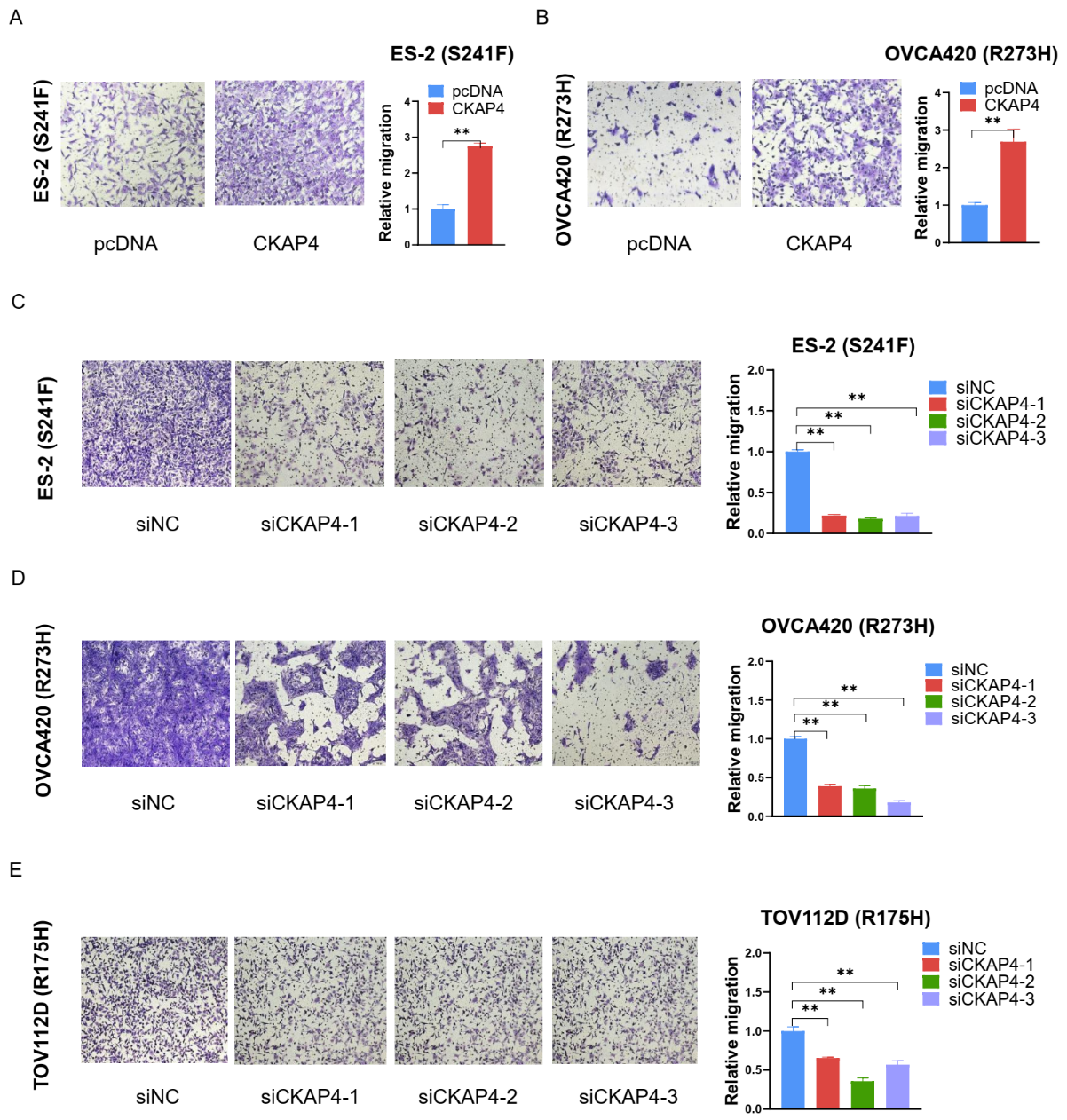


**Supplementary Figure 6. CKAP4 promotes ovarian cancer cell mobility.** (A, B) Overexpression of CKAP4 increases the migration of ES-2 and OVCA420 cells. Cells were transfected with control or CAKP4-encoding plasmid, followed by the cell migration assay. (C, D, E) Knockdown of CKAP4 reduces the migration of ES-2, OVCA420, and TOV112D cells. Cells were transfected with control or CAKP4 siRNAs, followed by the cell migration assay.


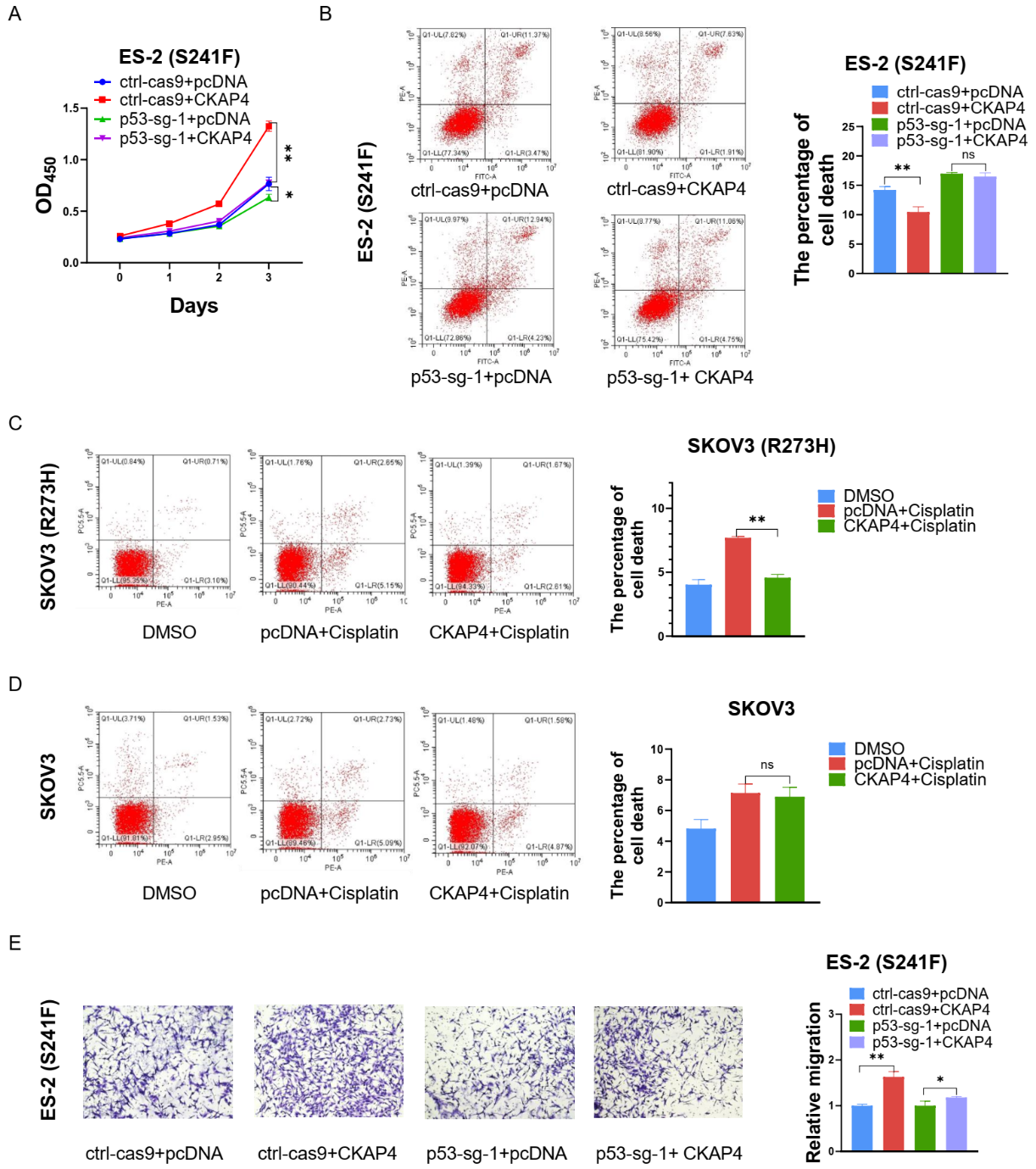


**Supplementary Figure 7. CKAP4 promotes ovarian cancer cell growth and migration partially dependent on mutant p53.** (A) Depletion of mtp53 impairs CKAP4’s ability to promote ovarian cancer cell proliferation. (B) Depletion of mtp53 impairs CKAP4’s ability to inhibit apoptosis of ovarian cancer cells. (C, D) CKAP4 overexpression inhibits cisplatin-induced apoptosis of mtp53-R273H-overexpressing SKOV3 cells, but not p53-null SKOV3 cells. (E) Depletion of mtp53 impairs CKAP4’s ability to promote ovarian cancer cell migration.


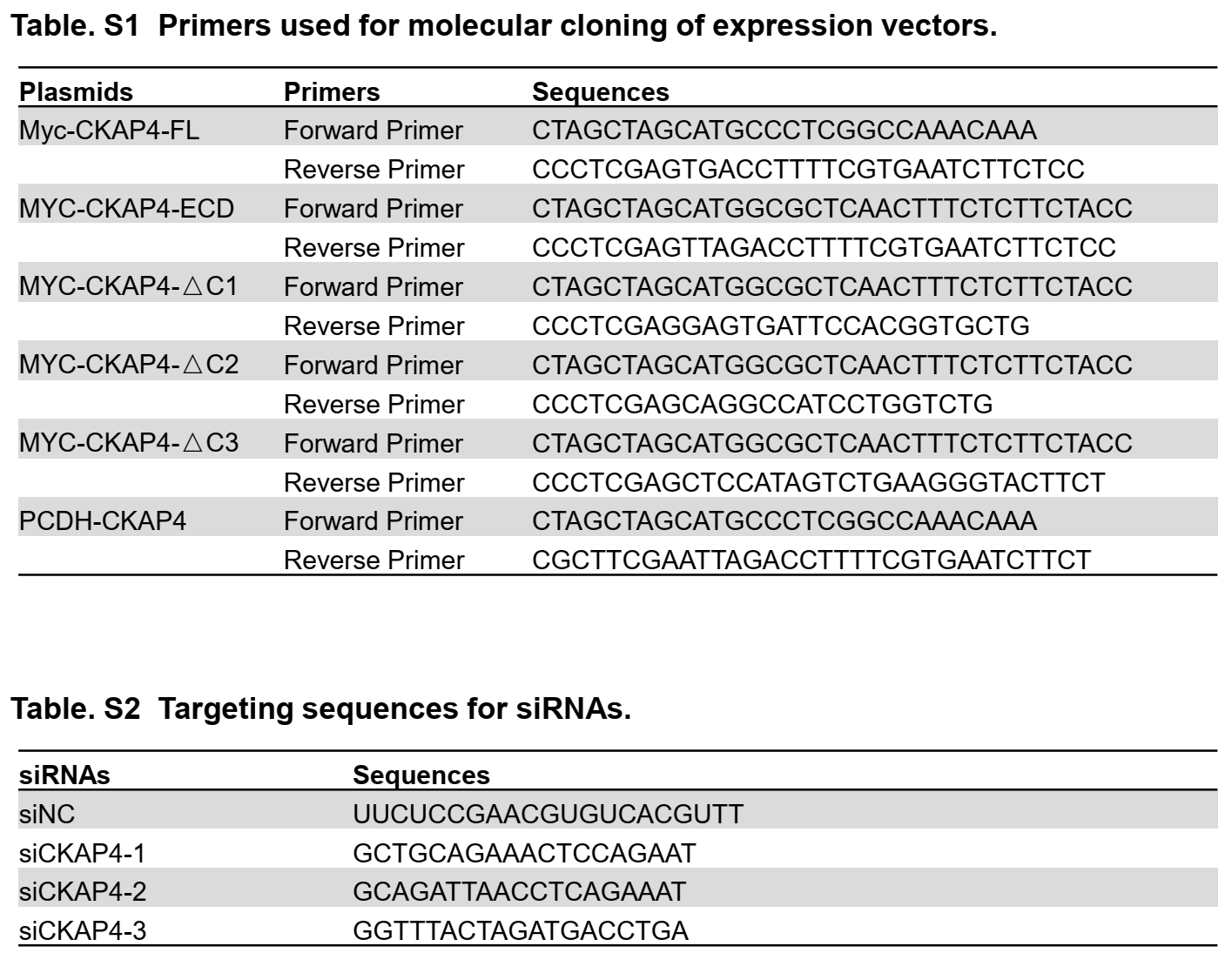


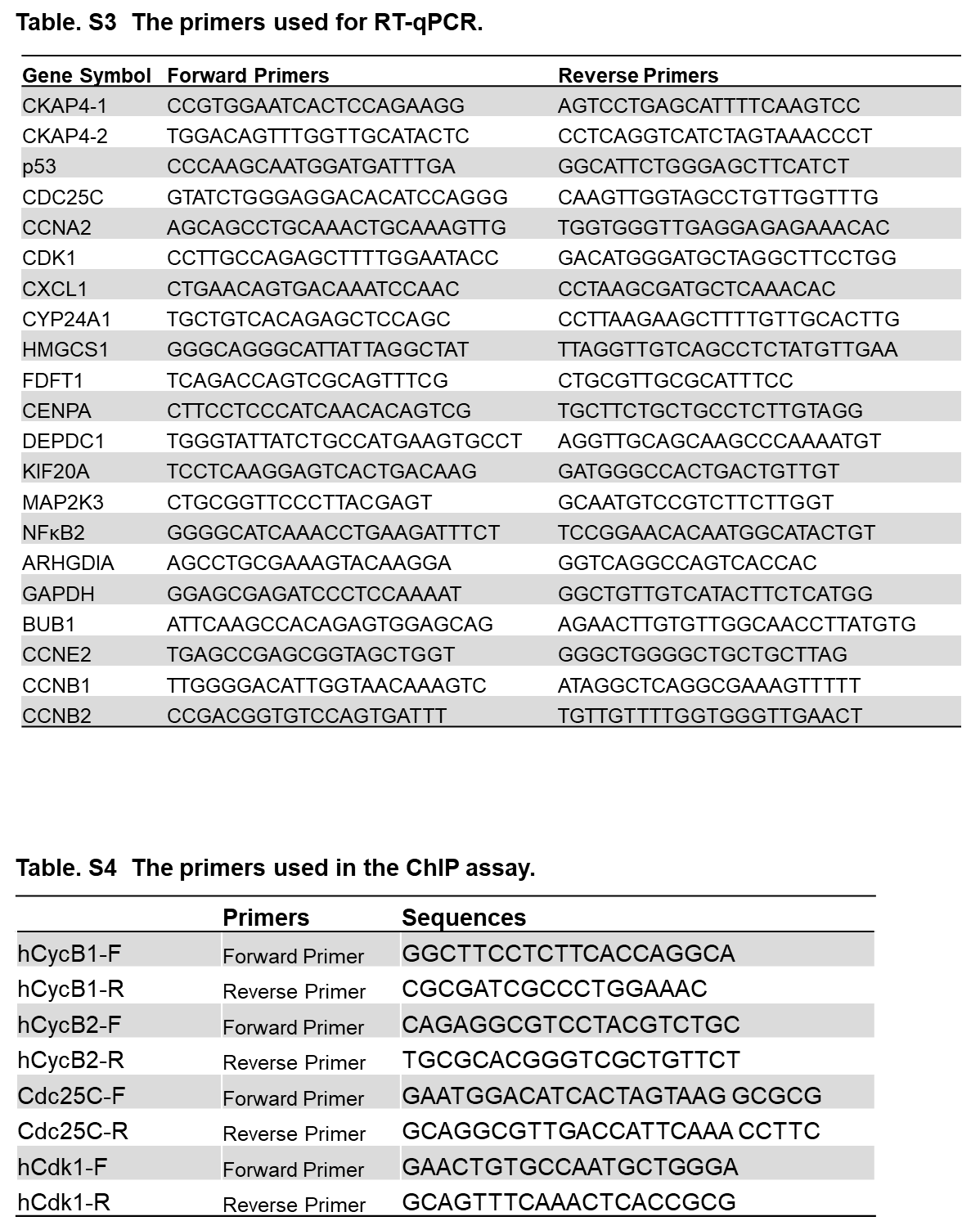

Supplement: Supplementary file 1 — Supporting information [file CTM2-13-e1476-s001.docx]
